# Aberrant activation of KRAS in mouse theca-interstitial cells results in female infertility

**DOI:** 10.3389/fphys.2022.991719

**Published:** 2022-08-19

**Authors:** Penghao Sun, Hongliang Wang, Lingyun Liu, Kaimin Guo, Xian Li, Yin Cao, Chemyong Ko, Zi-Jian Lan, Zhenmin Lei

**Affiliations:** ^1^ Department of Andrology, The First Hospital of Jilin University, Changchun, China; ^2^ Department of OB/GYN, University of Louisville School of Medicine, Louisville, KY, United States; ^3^ Department of Comparative Biosciences, University of Illinois at Urbana-Champaign, Urbana, IL, United States; ^4^ Birth Defects Center, University of Louisville School of Dentistry, Louisville, KY, United States

**Keywords:** KRAS, theca cell, Bmp7, ovulation, infertility

## Abstract

KRAS plays critical roles in regulating a range of normal cellular events as well as pathological processes in many tissues mediated through a variety of signaling pathways, including ERK1/2 and AKT signaling, in a cell-, context- and development-dependent manner. The *in vivo* function of KRAS and its downstream targets in gonadal steroidogenic cells for the development and homeostasis of reproductive functions remain to be determined. To understand the functions of KRAS signaling in gonadal theca and interstitial cells, we generated a *Kras* mutant (*tKrasMT*) mouse line that selectively expressed a constitutively active *Kras* ^*G12D*^ in these cells. *Kras* ^*G12D*^ expression in ovarian theca cells did not block follicle development to the preovulatory stage. However, *tKrasMT* females failed to ovulate and thus were infertile. The phosphorylated ERK1/2 and forkhead box O1 (FOXO1) and total FOXO1 protein levels were markedly reduced in *tKrasMT* theca cells. *Kras* ^*G12D*^ expression in theca cells also curtailed the phosphorylation of ERK1/2 and altered the expression of several ovulation-related genes in gonadotropin-primed granulosa cells. To uncover downstream targets of KRAS/FOXO1 signaling in theca cells, we found that the expression of bone morphogenic protein 7 (*Bmp7*), a theca-specific factor involved in ovulation, was significantly elevated in *tKrasMT* theca cells. Chromosome immunoprecipitation assays demonstrated that FOXO1 interacted with the *Bmp7* promoter containing forkhead response elements and that the binding activity was attenuated in *tKrasMT* theca cells. Moreover, *Foxo1* knockdown caused an elevation, whereas *Foxo1* overexpression resulted in an inhibition of *Bmp7* expression, suggesting that KRAS signaling regulates FOXO1 protein levels to control *Bmp7* expression in theca cells. Thus, the anovulation phenotype observed in *tKrasMT* mice may be attributed to aberrant KRAS/FOXO1/BMP7 signaling in theca cells. Our work provides the first *in vivo* evidence that maintaining normal KRAS activity in ovarian theca cells is crucial for ovulation and female fertility.

## Introduction

The Kirsten rat sarcoma viral oncogene homolog (*Kras*) is a member of three *Ras* (*Hras, Kras, and Nras*) family genes in mammals that encode a monomeric cytoplasmic protein with GTPase activity (Barbacid, 1987). It acts as a central component of the cellular networks communicating with extracellular stimuli through a variety of signaling pathways, including extracellular signal-regulated kinases 1 and 2 (ERK1/2) and phosphatidylinositol 3-kinase (PI3K)/protein kinase B (AKT) signaling, which modulate the growth, proliferation, survival, differentiation, adhesion, and motility of a cell (Malumbres and Barbacid, 2003; Han et al., 2021; Kim et al., 2021). Although *Kras* expression appears to be ubiquitous (Barbacid, 1987; Omerovic et al., 2007), its effects are cell-, context- and development-specific (Rocks et al., 2006; Sarkisian et al., 2007; Han et al., 2021). Studies of RAS-deficient mice reveal that *Kras* is the only member of the *Ras* gene family indispensable for embryonic development (Koera et al., 1997). *Kras* is the most frequently mutated in this family, and mutations are more likely at residue 12, resulting in a persistently GTP-bound form of KRAS and being inherently active independent of upstream input for activation (Bourne et al., 1990; Johnson et al., 2001). Constitutive activation of KRAS signaling is often associated with malignant cell transformation (Johnson et al., 2001; Murugan et al., 2019; Kim et al., 2021). *Kras* is expressed in various cell types of mammalian gonads (Fan and Richards, 2010; Tai and Ascoli, 2011; Fan et al., 2012; Richards et al., 2012). Amplified KRAS is observed in 11% of ovarian cancers and is much less common in testicular cancer cases (Cancer Genome Atlas Research, 2011; de Vries et al., 2020).

Mammalian gonads consist of three major cell populations, *i.e*., germ cells and steroidogenic and supporting somatic cells (Rotgers et al., 2018). The supporting cells, Sertoli cells in the testis and granulosa cells in the ovary, share a common precursor originating from the coelomic epithelium and play a vital role in nurturing the development of germ cells in the adult gonads. Sertoli cells support the progression of spermatogonia through meiosis to the production of motile spermatozoa. Granulosa cells, meanwhile, are a crucial component regulating the growth and maturation of ovarian follicles and the release of a fertilizable oocyte to produce an embryo. KRAS is an important physiological mediator of luteinizing hormone (LH) and follicle-stimulating hormone (FSH) signaling in granulosa cells (Fan and Richards, 2010; Fan et al., 2012). Abnormal activation of KRAS signaling in granulosa cells dysregulates the activity of ERK1/2 and AKT signaling and results in differentiation arrest of granulosa cells. The supporting cell-specific *Kras* mutant female mice are subfertile and display a phenotype resembling premature ovarian failure (Fan et al., 2008b). In contrast, mutant male mice are phenotypically healthy with grossly normal spermatogenesis and fertility (Richards et al., 2012).

Steroidogenic cells, interstitial cells in the testis and theca cells in the ovary, are crucial cell types in the gonadal interstitium that engage steroidogenesis and provide key hormones, androgens, for reproductive functions. They originate from the coelomic epithelium of the gonadal primordium and the neighboring mesonephros. Unlike testicular interstitial cells, ovarian theca cells do not differentiate during fetal gonadal development until perinatal stages when follicular development begins (Potter et al., 2016). Testicular androgen synthesis is obligatory for spermatogenesis and maintenance of the masculine phenotype. Androgens and many other growth regulatory factors produced by ovarian theca cells are important for normal follicle development, estrogen biosynthesis in granulosa cells, ovulation, and female fertility (Magoffin, 2005; Young and McNeilly, 2010; Richards et al., 2018). It is well characterized that androgenesis in theca cells is primarily regulated by LH signaling. While much has been learned about the factors that modulate steroidogenesis in theca cells, relatively less information is available about how the production of the factors in theca cells is regulated and what the roles are of these theca-produced factors, in addition to testosterone, in follicle development and ovulation. KRAS, a molecular switch in the transduction of multiple cellular signals, is known to be expressed in gonadal theca and interstitial cells (Shiraishi and Ascoli, 2006; Fan and Richards, 2010; Tai and Ascoli, 2011). However, the *in vivo* function of KRAS and its downstream targets in gonadal steroidogenic cells for the development and homeostasis of reproductive functions remain to be determined.

By knocking-in a constitutively active *Kras* specifically in mouse steroidogenic cells, the present study provides the first *in vivo* evidence that maintaining normal KRAS activity in ovarian theca cells is essential for ovulation and female fertility. Molecular analyses reveal that KRAS signaling in theca cells regulates forkhead box O1 (FOXO1) protein levels to control the expression of theca cell-specific growth factor bone morphogenic protein 7 (BMP7). Our results suggest that aberrant KRAS/FOXO1/BMP7 signaling in theca cells is likely an important cause of anovulation and female infertility.

## Materials and methods

### Animals and genotyping

Mice carrying a *lox-stop-lox (Lsl)* sequence followed by the *Kras* ^*G12D*^ point mutation allele (*LslKras* ^*G12D*^) (Johnson et al., 2001) were purchased from the Jackson Laboratories (Bar Harbor, ME, USA). *Cyp17iCre* mice (Bridges et al., 2008) were imported from the University of Kentucky (Lexington, KY, USA). *LslKras* ^*G12D*^ mice were crossed with *Cyp17iCre* mice to generate mice with theca-interstitial cell specific knocking-in of the *Kras* ^*G12D*^ mutant allele, hereafter referred to as *tKrasMT* mice. Mouse tail genomic DNA was extracted using ZR genomic DNA-tissue mini prep kits according to the procedure recommended by the manufacturer (Lan et al., 2017) (Zymo Research Corp, Irvine, CA, USA) and genotyped by PCR using the primers synthesized by Eurofins Genomics LLC (Louisville, KY, USA) as follows: 5′-*tgt​ctt​tcc​cca​gca​cag​t*-3’ (wild-type *Kras* allele forward), 5′-*gca​ggt​cga​ggg​acc​taa​ta*-3’ (*Kras* ^*G12D*^ mutant allele forward) and 5′-*ctg​cat​agt​acg​cta​tac​cct​gt*-3’ (common reverse for wild-type and mutant alleles).

All mice were on the C57BL/6 background and were housed on a 14-h light:10-h dark cycle with free access to food and water. The studies were approved by the Animal Care and Use Committee of the University of Louisville. All mice were sacrificed under ketamine anesthesia, and efforts were made to minimize their discomfort.

### Fertility, superovulation, and oocyte maturation studies

Six 7- to 8-week-old *tKrasMT* and control (*LslKras* ^*G12D*^) female mice were bred with proven fertile wild-type C57BL/6 males (1 male/1 female per cage) for 2 months. The total number of pups produced from each female was recorded as described previously (Lan et al., 2003).

Superovulation studies were performed on 21- to 25-day-old female littermates by intraperitoneal injections of 5 IU equine chorionic gonadotropin (eCG, Sigma, St Louis, MO, USA) for 48 h followed by 5 IU human chorionic gonadotropin (hCG, Sigma) for 22 h as described previously (Lan et al., 2003; Fan et al., 2009b). The total number of oocytes collected from both sides of the oviducts of each animal was recorded.

For the spontaneous oocyte maturation assay, immature oocytes were retrieved from 21- to 25-day-old eCG-primed (5 IU for 48 h) *tKrasMT* and control (*LslKras* ^*G12D*^) female mice according to previously described methods (Anderiesz et al., 2000; Lan et al., 2003). The oocytes were denuded by incubation with hyaluronidase solution immediately after removal from the ovarian follicle and cultured in 20 μL drops of Eagle’s Minimal Essential Medium–alpha modification (α-EMEM, Sigma) medium supplemented with 10% fetal bovine serum (FBS) under light mineral oil at 37°C for 24 h. The oocytes were examined with phase contrast microscopy for meiotic progression. The oocytes containing a polar body were considered *in vitro* matured.

### Histological and immunohistochemical staining

Ovaries and testes were fixed in 4% paraformaldehyde (Sigma) in phosphate-buffered saline (PBS) overnight and embedded in paraffin. Seven-micrometer-thick cross-sections were cut and used for hematoxylin and eosin (H&E) and immunohistochemical staining.

Immunohistochemistry was performed by an avidin-biotin immunoperoxidase method as described previously (Cao et al., 2022). Briefly, deparaffinized sections were rehydrated and then incubated with 1% H_2_O_2_ for 30 min. After rinsing with PBS and incubating with normal serum at room temperature (RT), the sections were incubated with rabbit anti-KRAS or anti-HSD3B1 (3-β-hydroxysteroid dehydrogenase 1) antibody (1:100, ABclonal, Woburn, MA, USA) overnight at 4°C and then incubated with biotinylated anti-rabbit IgG secondary antibody (1:100, Vector Laboratories, Burlingame, CA, USA) for 1 h at RT. After rinsing with PBS, sections were incubated with avidin-biotin-horseradish peroxidase complex using a Vectastain ABC kit (Vector Labs, Burlingame, CA, USA) for 1 h and rinsed with PBS. Immunostaining was detected by incubation of the sections with the substrate 3′3-diaminobenzidine at RT. All sections were counterstained with hematoxylin. Replacement of the primary antibody with irrelevant rabbit IgG was used as a procedure control.

### Isolation of granulosa, theca and interstitial cells

Ovaries isolated from eCG-primed 21- to 25-day-old female mice with the bursa removed were placed in RPMI 1640 medium (Sigma). Granulosa cells were collected by multiple follicle punctures using 30-gauge needles followed by filtration through 40-µm cell strainers (BD Falcon, Bedford, MA, USA) to remove oocytes. Theca cells were isolated as reported with minor modifications (Magoffin and Erickson, 1988; Li and Hearn, 2000). In brief, ovaries were punctured using 30-gauge needles to completely release antral granulosa/oocyte complexes. Punctured ovaries were incubated in 0.1°ml/ovary of 0.1% collagenase and 0.01% DNase I (Sigma) solution at 37°C to release theca cells. The incubation was terminated when theca layers from most preantral follicles were released into the solution, while most preantral granulosa/oocyte complexes remained intact (Fan et al., 2009a). Dispersed theca cells were obtained by filtration of the enzyme-digested solutions through 40-µm cell strainers (BD Falcon) to remove preantral granulosa/oocyte complexes and undigested ovarian tissues. The isolated theca cells were purified by a Percoll gradient centrifugation procedure. Briefly, dispersed cells were layered on top of a discontinuous Percoll density gradient (a specific gravity of 1.055 and 44% Percoll solution, Sigma) and then centrifuged for 20 min at 400 X g. The cells within the gravity of 1.055 Percoll layer were collected (Magoffin and Erickson, 1988; Li and Hearn, 2000). RT-PCR tests showed that isolated cells expressed high levels of theca cell marker genes (*Lhcgr*, *Hsd3b1* and *Cyp17b1*) but not granulosa cell marker genes (*Fshr* and *Cyp19a1*).

Interstitial cells from the testes of adult *tKrasMT* mice were prepared as described by Yang *et al* (Yang et al., 2016). In brief, the testes were decapsulated, cut into small pieces, and then subjected to a gentle mechanical dissociation followed by a 15-min digestion at 34°C with 0.05% collagenase (Sigma). The crude testicular cell suspension obtained was then filtered through 70-µm cell strainers (BD Falcon), and the cells were layered on top of discontinuous Percoll gradients (5–70%, Sigma) and centrifuged.

### Reverse transcription-polymerase chain reaction and quantitative RT-PCR

Total RNA from testes, interstitial cells, ovaries, adrenal glands, uteri, pituitaries, theca cells, granulosa cells and oocyte-granulosa mixtures was isolated using TRIzol reagent (Invitrogen, Carlsbad, CA, USA) as described previously (Yuan et al., 2010). Briefly, total RNA was adjusted to a concentration of approximately 1.0 μg/μL. Two microgram of total RNA was reverse transcribed into cDNA with random primers (Invitrogen) and avian myeloblastosis virus (AMV) reverse transcriptase (Promega Corp, Madison, WI, USA). *Kras* ^*G12D*^ expression in the ovary, theca cells, granulosa cells, interstitial cells and testes was detected by RT-PCR using the primer set (5′-*agg​cct​gct​gaa​aat​gac​tg*-3′ and 5′-*ccc​tcc​cca​gtt​ctc​atg​ta*-3′, Eurofins Genomics LLC) followed by digestion of amplified PCR products with Hind III (Promega Corp) as described (Aguirre et al., 2003). The digested products were separated by electrophoresis in agarose gels and stained with ethidium bromide. The *Kras* ^*G12D*^ allele, but not the wild-type *Kras* allele, contained a Hind III restriction site engineered in exon 1. Digestion of the 243°bp PCR products with Hind III yielded 213 and 30 base pair bands from the mutant products only.

qRT-PCR analyses were performed as described previously (Lan et al., 2003). In brief, first strand cDNA was synthesized using TaqMan RNA reverse transcription reagents (Thermo Fisher Scientific, Waltham, MA, USA). Predesigned TaqMan probes and primers for *Dusp6* (Duel-specific phosphatase 6, Mm00518185_m1), *Foxo1* (Forkhead box O1, Mm00490672_m1), *Bmp7* (Bone morphogenetic protein 7, Mm00432102_m1), *Fshr* (FSH receptor, Mm00442819_m1), *Hsd17b1* (Hydroxysteroid 17-Beta-dehydrogenase 1, Mm00501692_g1), *Cyp19a1* (Aromatase, Mm00484049_m1), *Cyp11a1* (Cytochrome P450 cholesterol side-chain cleavage enzyme, Mm00490735_m1), *Nrip1* (Nuclear receptor interacting protein 1, Mm00476537_s1), *Pten* (Phosphatase and tensin homolog, Mm00477208_m1), *Npr2* (Natriuretic peptide receptor 2, Mm00612889_m1), *Areg* (Amphiregulin, Mm01354389_m1) and *Btc* (Betacellulin*,* Mm00432137_m1) were purchased from Applied Biosystem/Thermo Fisher Scientific. Our previously reported TaqMan probes and primers of *Star* (Steroidogenic acute regulatory protein), *Cyp17a1* (Cytochrome P450 family 17 subfamily A member 1), *Lhcgr* (LH/hCG receptor)*, Hsd3b1* (Hydroxy-delta-5-steroid dehydrogenase) and *Actb* (Actin beta) were used to determine their expression in the samples (Lan et al., 2003). Relative mRNA levels were obtained after normalization to the mRNA levels of the housekeeping gene *Actb* in each sample. At least three individual RNA samples per group were examined.

### Hormone assays

Eleven-to 12-week-old male mice and female mice at diestrous were anesthetized and exsanguinated by cardiac puncture. Sera were separated and stored at −80°C until assayed. FSH, estradiol and testosterone levels were determined by the Ligand Assay and Analysis Core Center for Research in Reproduction, the University of Virginia (Charlottesville, VA, USA). Mouse FSH, estradiol and testosterone levels were measured by radioimmunoassay (RIA). The limits of sensitivities of these assays were 2 ng/ml for FSH, 10 pg/ml for estradiol and 5 ng/dl for testosterone.

### Theca cell culture, *Foxo1* knockdown and *pCMV-Foxo1* transfection

The isolated theca cells were maintained in 12-well plates with RPMI-1640 medium containing 25 mM HEPES, 2 mM l-glutamine, 10% heat-inactivated FBS and 1% Pen-Strep (Invitrogen). Cells were then cultured at 37°C in an incubator with 95% air and 5% CO_2._ For *Foxo1* knockdown, theca cells were isolated from 60-day-old wild-type ovaries and transfected with *Foxo1* siRNA using a procedure described previously (Yuan et al., 2010). The *Foxo1* siRNAs, purchased from Santa Cruz Biotech (Dallas, TX, USA) consisted of a pool of 3–5 target-specific siRNAs against mouse *Foxo1*. Typically, a mixture of 0.25 μg siRNA, 100 μL RPMI-1640 and 4 μL Lipofectamine 2000 (Invitrogen) was added to the cells in a 12-well plate and incubated overnight. The transfection medium was replaced with 0.5 ml RPMI-1640 containing 10% FBS (Sigma) for an additional 24°h, and these cells were used for subsequent experiments. A control siRNA (Santa Cruz Biotech) consisting of a scrambled RNA sequence was transfected in parallel with *Foxo1* siRNA as a specificity control for the knockdown. Western blotting was used to determine the effectiveness of *Foxo1* siRNAs in suppressing FOXO1 protein levels in theca cells. A transient DNA transfection procedure described previously was used to overexpress mouse *Foxo1* in *tKrasMT* theca cells (Cao et al., 2022). Briefly, the cells were cultured to approximately 80% confluence in 12-well plates and were transiently transfected with 1 µg of *pCMV-Foxo1* plasmid DNA (Nakae et al., 2001) (Addgene, Watertown, MA, USA) with 2 µL of X-TremeGene 360 reagent (Roche, Mannheim, Germany) per well for 72 h. The cells were then cultured in RPMI-1640 containing 10% FBS. The cells transfected with empty vector *pCMV* served as the control. FOXO1 protein levels in transfected cells were checked by Western blot.

### Western blotting

Granulosa and theca cells were homogenized by sonication with a sonic dismembrator Model 150 (Thermo Fisher Scientific) in ice-cold lysis buffer containing complete proteinase and phosphatase inhibitor cocktails (Roche, Indianapolis, IN, USA). The protein concentrations were measured by the Bradford method (Bio-Rad Laboratories, Hercules, CA, USA). Five μg/lane of cell lysates were separated in SDS-PAGE gels, transferred to Immobilon-PVDF membranes (Millipore, Billerica, MA, USA), blocked with 3% nonfat milk, and then incubated overnight with primary antibodies. Phospho-ERK1/2^T202/Y204^, phospho-AKT^S473^, AKT, phospho-CREB^S133^, CREB, phospho-FOXO1^S256^ and FOXO1 antibodies were procured from Cell Signaling Technology Inc. (Danvers, MA, USA). ERK1/2 and β-actin antibodies were purchased from Sigma. Peroxidase-conjugated secondary antibody (1:2,000, Vector Laboratories) was used as the secondary antibody. Immunoblotting signals were detected by the enhanced chemiluminescence (ECL) Western blotting detection system (GE Healthcare Biosciences, Pittsburgh, PA, USA) as described previously (Li et al., 2016). All membranes were reblotted with β-actin antibody (1:1,000) as the loading control. The intensity of specific bands was scanned and semiquantified using the image analysis software, TotalLab V (Nonlinear USA Inc., Durham, NC). The optical density of the protein bands was normalized to that of β-actin. Experiments were repeated three times on samples from at least three animals.

### Chromatin immunoprecipitation

The ChIP assays were performed using an Imprint Ultra ChIP kit (Sigma) as described previously (Lin et al., 2014; Lin and Lei, 2016). Briefly, theca cells isolated from 60-day-old ovaries were cultured with RPMI-1640 medium supplemented with 10% FBS. The chromatin was crosslinked by treating the cells with 1% formaldehyde for 10 min at room temperature. The cells were harvested and homogenized with a glass Dounce homogenizer (Thermo Fisher Scientific). The crosslinked chromatin was fragmented by sonication (3 × 10 min) with a sonic dismembrator Model 150 (Thermo Fisher Scientific). Rabbit anti-FOXO1 antibody (Cell Signaling Technology) at 1:100 dilution was used for immunoprecipitation. The DNA was then purified and used for PCR with the primer set (5′-*ggc​agg​aga​atc​tct​gtg​aac​t*-3′ and 5′-*aac​att​cag​cta​agg​tgc​caa​g*-3′, Eurofins Genomics LLC) and the conditions (denaturation for 30 s at 95°C, annealing for 30 s at 60°C, and extension for 1 min at 72°C.) for 40 cycles. The PCR products that amplified the *Bmp7* promoter from the -5,465 to -5,261 bp region were validated by Pst I (Promega) digestion.

### Statistical analysis

The data presented are the means ± SEM of three samples or indicated numbers of samples in the figures. The results were analyzed by one-way analysis of variance (ANOVA) and unpaired Student’s t test using a version 3.06 Instat program (GraphPad Software, San Diego, CA). A *p* value < 0.05 was considered statistically significant.

## Results

### Generation of *tKrasMT* mice

**FIGURE 1:**
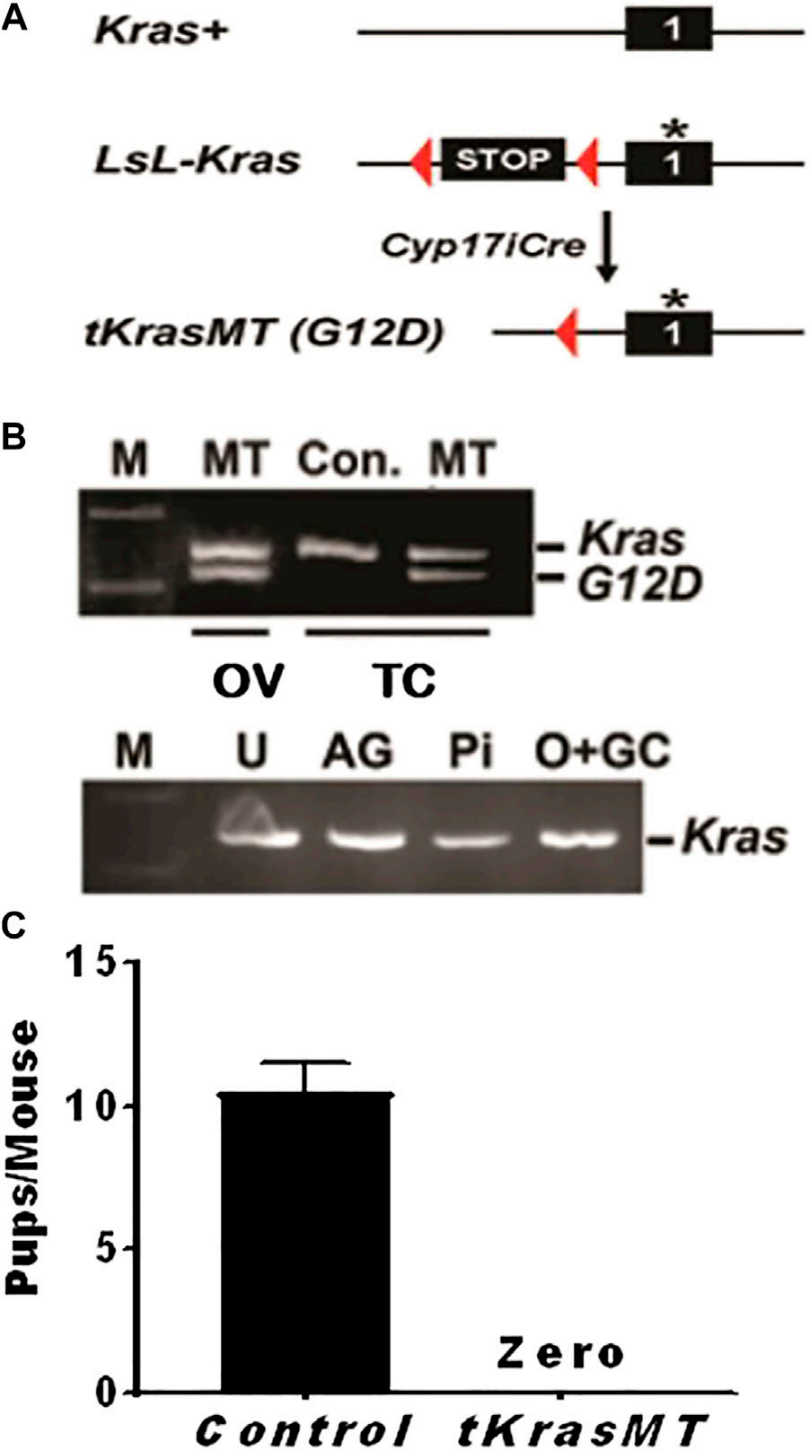
Knocking in a constitutively active *Kras* (*Kras* ^*G12D*^) in theca cells (*tKrasMT*) results in female infertility. **(A)** A *Cre/loxP* strategy for the generation of *tKrasMT* mice. An asterisk represents a G12D mutation in KRAS protein. **(B)**. RT-PCR showing *Kras* ^*G12D*^ expression in *tKrasMT* ovaries (OV) and theca cells (TC) but not in uteri (U), adrenal glands (AG), pituitary (Pi), or oocyte and granulosa cell mixtures (O + GC) of *tKrasMT* mice. *Kras* ^*G12D*^ expression is visualized by the digestion of PCR products with Hind III that has been introduced into the *Kras* ^*G12D*^ exon. **(C)**. No pups were produced by mature *tKrasMT* female mice during the 2-months breeding assay (n = 6).

Immunohistochemical staining demonstrated that KRAS was expressed in multiple cell types in gonads, including theca and granulosa cells as well as oocytes in the ovaries (Supplementary Figures S1A,B) and interstitial and spermatogenic cells in the testes (Supplementary Figures S1C,D). By crossing *lox-stop-lox-Kras* ^*G12D*^ *(LslKras* ^*G12D*^ *)* mice (Johnson et al., 2001) with previously generated *Cyp17iCre* transgenic mice that express *iCre* in theca and interstitial cells of ovaries and testes (Bridges et al., 2008) (Figure 1A), *LslKras* ^*G12D*^ *;Cyp17iCre* mice were generated and named *tKrasMT* mice. *tKrasMT* male and female mice survived to adulthood.

To determine whether *Kras* ^*G12D*^ was expressed in the ovaries of *tKrasMT* mice, total RNA was isolated from the ovaries, uteri, adrenal glands, and pituitaries of 4-week-old *tKrasMT* mice and then subjected to RT-PCR followed by Hind III digestion of PCR products as described (Aguirre et al., 2003). As shown in Figure 1B, *Kras* ^*G12D*^ transcripts were detected only in the ovaries but not in the uteri, adrenal glands, or pituitaries of *tKrasMT* mice. To determine whether *Kras* ^*G12D*^ was selectively expressed in theca cells of *tKrasMT* ovaries, theca cells and oocyte-granulosa mixtures were isolated from eCG-primed 21- to 25-day-old *tKrasMT* females and then subjected to the above described *Kras* ^*G12D*^ expression analysis. As shown in Figure 1B, *Kras* ^*G12D*^ transcripts were detected in purified theca cells but not oocyte-granulosa mixtures of *tKrasMT* mice.

### Indistinguishable testicular phenotype of *tKrasMT* from *LslKras* ^*G12D*^ male mice

RT-PCR demonstrated *Kras* ^*G12D*^ expression in interstitial cells of the testis in *tKrasMT* males but not in *LslKras* ^*G12D*^ control mice (Supplementary Figure S2A). *tKrasMT* male mice were phenotypically normal and had grossly unaltered fertility and spermatogenesis. The testis, epididymis (Supplementary Figures S2B,C) and other sex accessory organs in *tKrasMT* males were indistinguishable from those in *LslKras* ^*G12D*^ controls. Adult *tKrasMT* males sired normally (data not shown). Histological examination of adult testes and epididymis revealed normal spermatogenesis in *tKrasMT* males (Supplementary Figures S2D,E,H,I). There was no difference in the immunostaining intensity of HSD3B1, a critical androgen biosynthesis enzyme, in interstitial cells between *tKrasMT* mice and *LslKras* ^*G12D*^ controls (Supplementary Figures S2F,G). Since no overt testis or fertility phenotypes were observed in *tKrasMT* males, this report focused on the characterization of ovarian and fertility phenotypes of *tKrasMT* females.

### Female sterility and the absence of corpous leteum in adult *tKrasMT* female mice

To determine whether there is any fertility defect in *tKrasMT* female mice, six 6-week-old *tKrasMT* females and six *LslKras* ^*G12D*^ control females were bred with stud males for 2 months. Within the breeding period, all six controls produced two litters of progenies with an average of 10.5 total pups (±1.0) per mouse (Figure 1C). However, *tKrasMT* females produced no progeny (Figure 1C). Thus, *tKrasMT* mice displayed female sterility.

**FIGURE 2:**
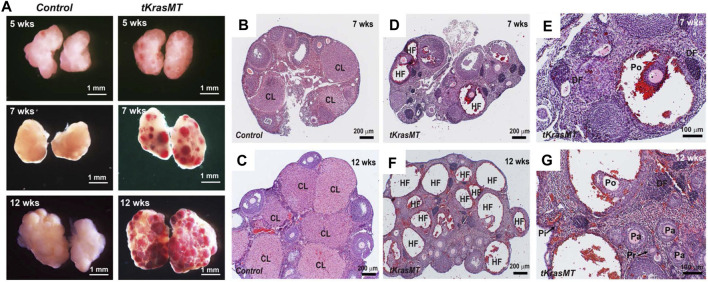
Ovarian phenotype of *tKrasMT* females. **(A)**. Gross ovarian morphology of 5-, 7- and 12-week-old females shows age-dependent ovary hemorrhage in *tKrasMT* mice. **(B–G)**. Ovarian histology of 7- and 12-week-old controls [*LslKras* ^*G12D*^ **(B,C)]** and *tKrasMT* **(D–G)** mice. Follicle hemorrhage and degeneration are obvious, but the corpus luteum is absent in adult *tKrasMT* ovaries. Panels E and G are higher magnifications of Panels D and F, respectively. CL, corpus luteum; HF, hemorrhagic follicles; DF, degenerating follicles; Pi, primordial follicle; Pr, primary follicle; Pa, preantral follicle; Po, preovulatory follicles. Note the dislodgement of peripheral mural granulosa cells in large antral/preovulatory follicles in F and G to form a cyst-like structure.

To gain insight into the cause of female sterility in *tKrasMT* mice, we examined the gross morphology and histology of ovaries from *LslKras* ^*G12D*^ control and *tKrasMT* mice at various ages. At the age of 5 weeks, there was no difference in the gross morphology (Figure 2A) or weight of ovaries between controls and *tKrasMT* mice. The ovarian weight (mg)/body weight (g) was 0.296 ± 0.042 (control, n = 6) *vs.* 0.306 ± 0.002 (*tKrasMT*, n = 6, Supplementary Figure S3A). However, hemorrhagic ovaries without visible corpora lutea were observed in 7- or 12-week-old *tKrasMT* ovaries (Figure 2). Histological studies on serial sections (Figures 2B–G) showed that follicles at each developmental stage (from primordial to preovulatory stage) were present in *tKrasMT* ovaries despite the presence of some degenerating preantral follicles (Figures 2D–G). Interestingly, follicles at the late antral and preovulatory stages in adult *tKrasMT* mice were hemorrhagic with visible loss or dislodgement of peripheral mural granulosa cells to form a cyst-like structure (Figures 2D,F). Strikingly, no corpora lutea were found in adult *tKrasMT* ovaries (Figures 2E,G). Ovarian tumors and hyperplasia in ovarian cells, including theca cells, were not observed in 3- to 12-week-old *tKrasMT* mice examined.

### Anovulation and no corpus luteal formation in immature *tKrasMT* mice in response to exogenous gonadotropins

**FIGURE 3:**
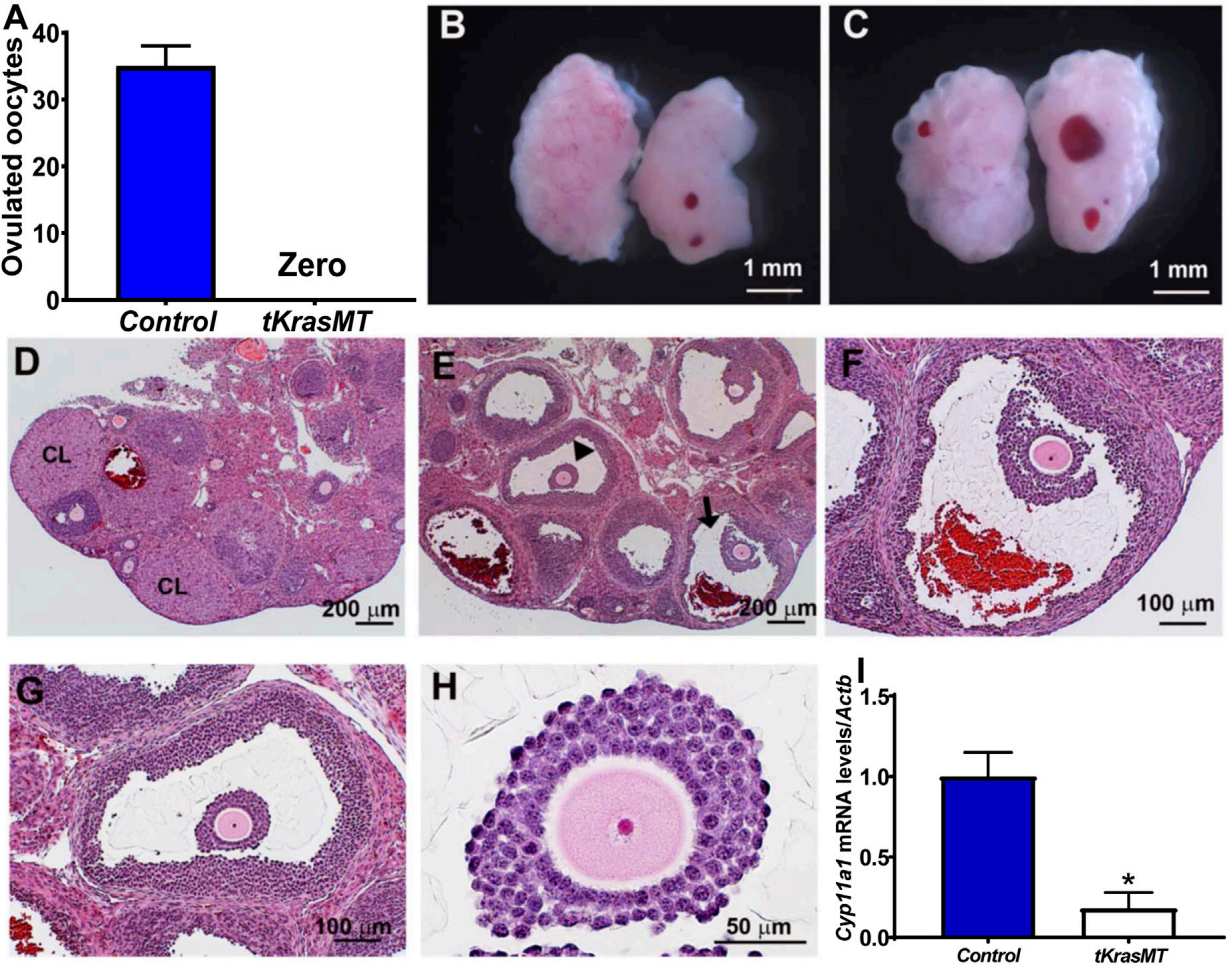
Anovulation and no corpus luteum formation in immature *tKrasMT* mice primed with exogenous gonadotropins. Numbers of ovulated oocytes (n = 6) **(A)**, gross morphology **(B,C)** and histology **(D–H)** of ovaries in immature control [*LslKras* ^*G12D*^ **(B,D)]** and *tKrasMT* **(C,E–H)** mice treated with eCG for 48 h followed by hCG for 22 h. Panels F & G are a higher magnification of follicles indicated by an arrowhead and an arrow in Panel E, respectively. Panel H is a higher magnification of a preovulatory follicle in *tKrasMT* ovary in Panel G showing a lack of cumulus expansion in response to exogenous gonadotropins. **(I)**. Reduced *Cyp11a1* mRNA levels in *tKrasMT* ovaries by qRT-PCR. *, *p* < 0.01 compared to the control (*Lslkras* ^*G12D*^) group (n = 6).

The above morphological and histological studies on adult *tKrasMT* ovaries indicate that *tKrasMT* mice likely have a defect in ovulation. To investigate this possibility and to avoid the complexity of endocrine hormones in adult mice for ovulation, immature (3- to 4-week-old) *LslKras* ^*G12D*^ control and *tKrasMT* female mice were subjected to superovulation studies. As shown in Figure 3A, immature *tKrasMT* females failed to ovulate in response to exogenous gonadotropin stimulation. Gross morphological analyses showed that there was no difference between *LslKras* ^*G12D*^ control (Figure 3B) and *tKrasMT* (Figure 3C) mice after eCG/hCG treatments. Ovarian weight (mg)/body weight (g) was 0.729 ± 0.088 (control, n = 5) vs. 0.788 ± 0.089 (*tKrasMT*, n = 5, Supplementary Figure S3A). Unlike *LslKras* ^*G12D*^ controls (Figure 3D), there was no disruption of follicle development to the preovulatory stage in immature *tKrasMT* mice after gonadotropin treatments. No corporus luteum and no sign of germinal vesicle breakdown, cumulus expansion, or follicle rupture were observed in the ovaries of gonadotropin-treated immature *tKrasMT* mice (Figures 3E–H). To further confirm the absence of corporus luteum in eCG/hCG-treated *tKrasMT* mice, qRT-PCR was performed to examine the expression of a healthy corpus luteal marker gene, *Cyp11a1*. As shown in Figure 3I, *Cyp11a1* mRNA levels were markedly lower in the ovaries of gonadotropin-treated *tKrasMT* mice than in those of *LslKras* ^*G12D*^ control mice.

*In vitro* oocyte maturation analysis showed that denuded oocytes from eCG primed prepuberal *tKrasMT* female mice*,* the same as the controls, were capable of progressing to metaphase II (Supplementary Figure S3B), indicating that the *tKrasMT* oocytes were competent for spontaneous maturation.

### Downregulation of forkhead box O1 protein levels and enhanced *Bmp7* transcription in *tKrasMT* theca cells

**FIGURE 4:**
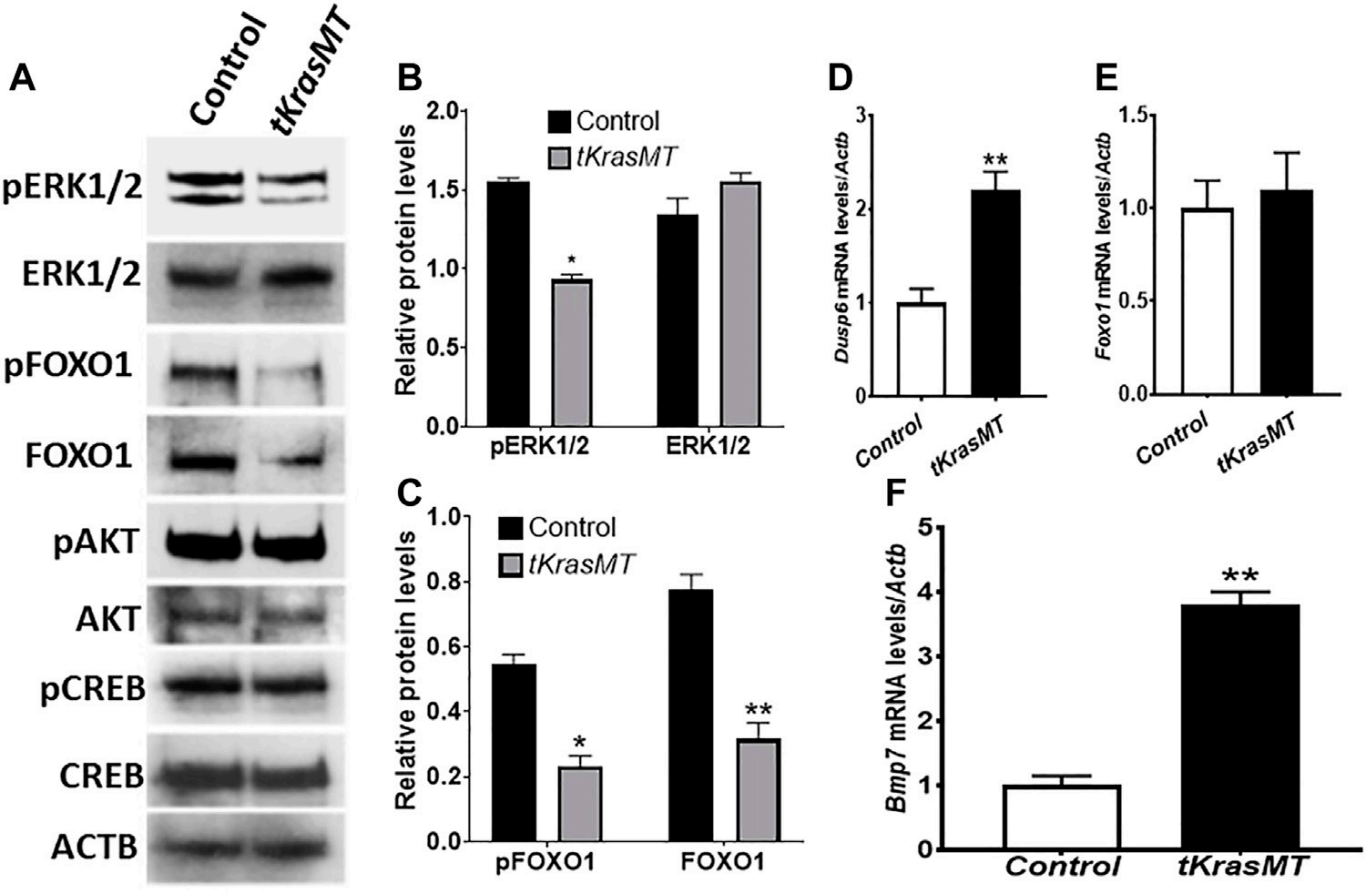
Reduced pERK1/2 and FOXO1 protein levels and elevated Bmp7 expression in *tKrasMT* theca cells. **(A)**. Expression of total and phosphorylated proteins of various signaling molecules in control (*LslKras* ^*G12D*^) and *tKrasMT* theca cells. Theca cell protein extracts were collected from 3-5 immature control or *tKrasMT* mice pretreated with eCG for 48 h, and subjected to Western blot analyses. Equal amounts of proteins (5 µg proteins) were loaded in each lane, and data were obtained from the same membrane blot using the indicated antibodies. **(B,C)**. Western blot results show that the relative protein levels of pERK1/2 **(B)**, pFOXO1 and FOXO1 **(C)** but not ERK1/2 **(B)** are significantly reduced in *tKrasMT* theca cells. **(D)**. Increased *Dusp6* expression in *tKrasMT* theca cells by qRT-PCR. **(E)**. No significant change in *Foxo1* mRNA expression in *tKrasMT* theca cells by qRT-PCR. **(F)**. Elevated *Bmp7* mRNA expression in *tKrasMT* theca cells by qRT-PCR. *, *p* < 0.05; **, *p* < 0.01 compared to the control (*Lslkras* ^*G12D*^) group (n = 5).

To investigate whether *Kras* ^*G12D*^ regulates MAPK, PI3K/AKT and PKA signaling in theca cells, theca cells were isolated from eCG-primed *LslKras* ^*G12D*^ control and *tKrasMT* mice, and subjected to Western blot analysis of their representative signaling molecules. As shown in Figure 4, the levels of phospho-ERK1/2 (pERK1/2, indicators of MAPK signaling), but not total ERK1/2, were reduced in *tKrasMT* theca cells (Figures 4A,B). In contrast, phospho-AKT (pAKT, an indicator of PI3K/AKT signaling), phospho-CREB (pCREB, an indicator of PKA signaling) and their total protein levels were not obviously altered in *tKrasMT* theca cells (Figure 4A). Downregulation of pERK1/2 levels has been observed in ovarian granulosa cells and nongonadal cells under the condition of persistent activation of RAS proteins, including KRAS^G12D^, and this is caused by the induced expression of phosphatases such as *Dusp6* (Camps et al., 2000). To investigate this possibility in theca cells, qRT-PCR was performed to examine *Dusp6* expression in *tKrasMT* theca cells. As shown in Figure 4D, *Dusp6* expression was significantly elevated in *tKrasMT* theca cells. Together, KRAS^G12D^ in theca cells mainly influenced ERK1/2 signaling with minimal effects on AKT or PKA signaling.

Reduced pERK1/2 in theca cells of *tKrasMT* ovaries prompted us to examine *Foxo1* expression at both the protein and mRNA levels. As shown in Figures 4A,C, total FOXO1 and phospho-FOXO1 (pFOXO1) protein levels were markedly decreased in *tKrasMT* theca cells. However, *Foxo1* mRNA expression was not significantly altered in *tKrasMT* theca cells (Figure 4E).

FOXO1 is a transcription factor that binds forkhead responsive elements (5′-(G/C)(T/A)AA(C/T)AA-3′, FHREs**)** to regulate gene expression (Furuyama et al., 2000; Huang and Tindall, 2007). Reduced FOXO1 protein levels in *tKrasMT* theca cells suggest that FOXO1-regulated gene(s) exist in theca cells, which are involved in the regulation of ovulation or corpus luteal formation. To identify the potential FOXO1 target gene(s), qRT-PCR analyses were performed on isolated *tKrasMT* theca cells. As shown in Figure 4F, the expression of *Bmp7,* a theca-specific growth factor (Shimasaki et al., 1999; Erickson and Shimasaki, 2003), was significantly elevated in *tKrasMT* theca cells. In contrast, the expression of *Star*, *Cyp11a1, Hsd3b1, Cyp17a1* and *Hsd17b1*, which are critical for steroidogenesis, was not significantly altered in *tKrasMT* theca cells (Supplementary Figure S4D). In addition, sera were also collected from adult *tKrasMT* mice at diestrus for steroid hormone analyses. The results showed that serum testosterone levels were not significantly altered in *tKrasMT* mice (Supplementary Figure S4A).

### Indirect influences of KRAS^G12D^ on *Areg, Btc*, *Cyp19a1* and *Cyp11a1* expression in preovulatory granulosa cells

**FIGURE 5:**
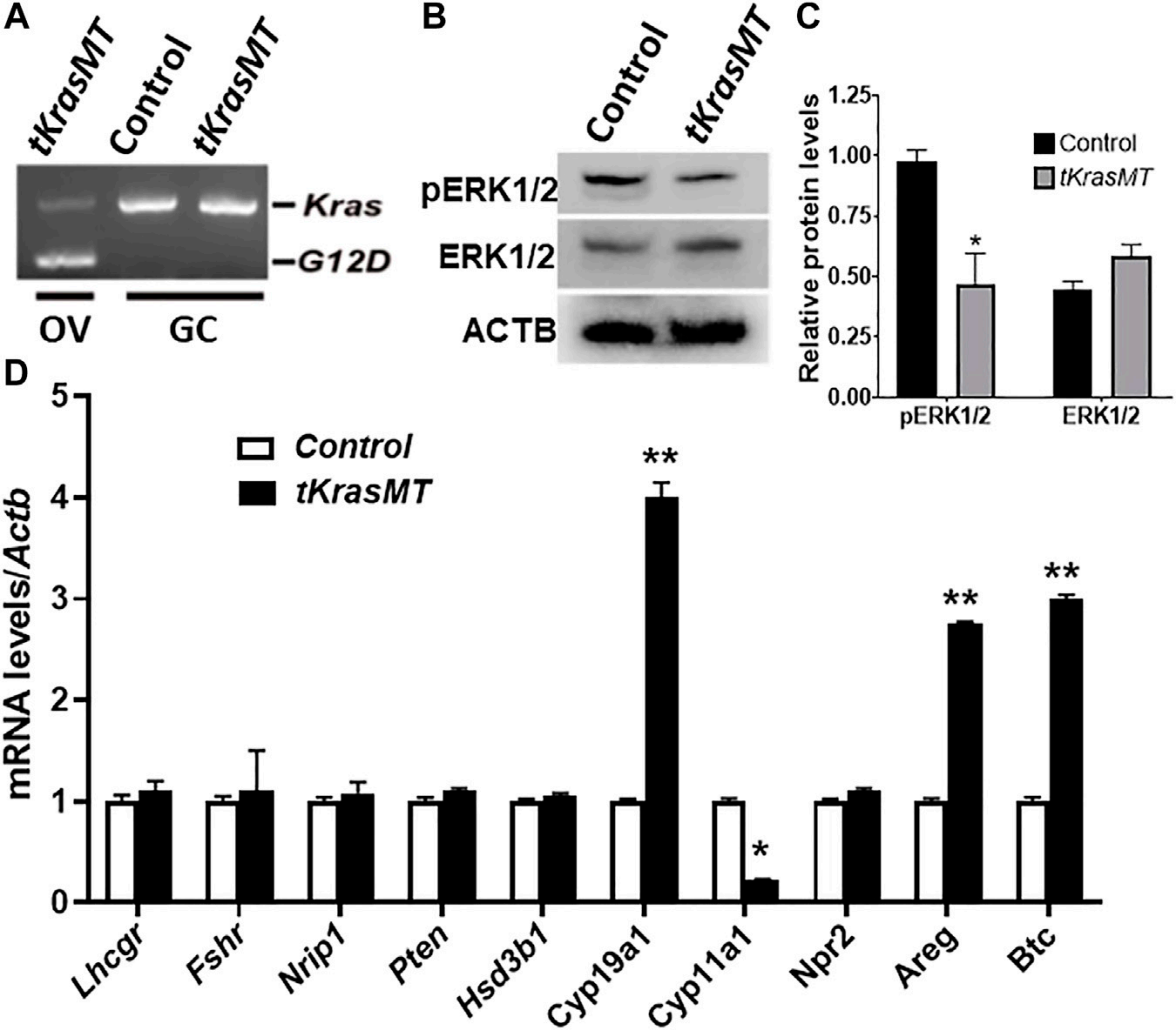
Granulosa cells of *tKrasMT* mice. **(A**). RT-PCR showing the absence of *Kras* ^*G12D*^ expression in preovulatory granulosa cells (GC) isolated from eCG-primed immature *tKrasMT* mice. **(B,C)**. Western blot results show that the relative protein levels of pERK1/2 but not ERK1/2 are markedly reduced in *tKrasMT* granulosa cells. **(D)**. Misexpression of genes associated with ovulation and oocyte maturation and/or cumulus expansion in preovulatory granulosa cells from *tKrasMT* mice by qRT-PCR. *, *p* < 0.05; **, *p* < 0.01 compared to the control (*Lslkras* ^*G12D*^) group (n = 5).

To investigate whether there exists a paracrine regulation of granulosa cell function from theca cells in *tKrasMT* mice, preovulatory granulosa cells were isolated from eCG-treated control and *tKrasMT* mice and subjected to qRT-PCR analyses of several genes involved in ovulation processes, estradiol biosynthesis and granulosa cell differentiation. As expected, *Kras* ^*G12D*^ was not expressed in isolated preovulatory granulosa cells of *tKrasMT* mice (Figure 5A). Western blot results revealed a reduction in the phosphorylation of ERK1/2 in preovulatory granulosa cells (Figures 5B,C). Analyses of genes involved in the ovulation process showed that the expression of *Areg* and *Btc* but not *Nrip1*, *Npr2* and *Pten* was markedly increased in *tKrasMT* granulosa cells (Figure 5D). In addition, *Fshr*, *Lhcgr*, and *Hsd3b1* were not significantly altered in preovulatory granulosa cells from *tKrasMT* mice (Figure 5D). In contrast, the expression of *Cyp19a1* was significantly increased, while the expression of *Cyp11a1* was markedly decreased in *tKrasMT* granulosa cells. In addition, sera of adult *tKrasMT* mice were also collected and subjected to RIA analyses of estradiol and FSH levels. Serum estradiol but not FSH levels were significantly elevated in *tKrasMT* mice (Supplementary Figures S4B,C). Together, *Kras* ^*G12D*^ in theca cells indirectly caused mis-expression of *Areg, Btc*, *Cyp19a1* and *Cyp11a1* in preovulatory granulosa cells.

### Regulation of *Bmp7* expression by FOXO1 in *tKrasMT* theca cells

**FIGURE 6:**
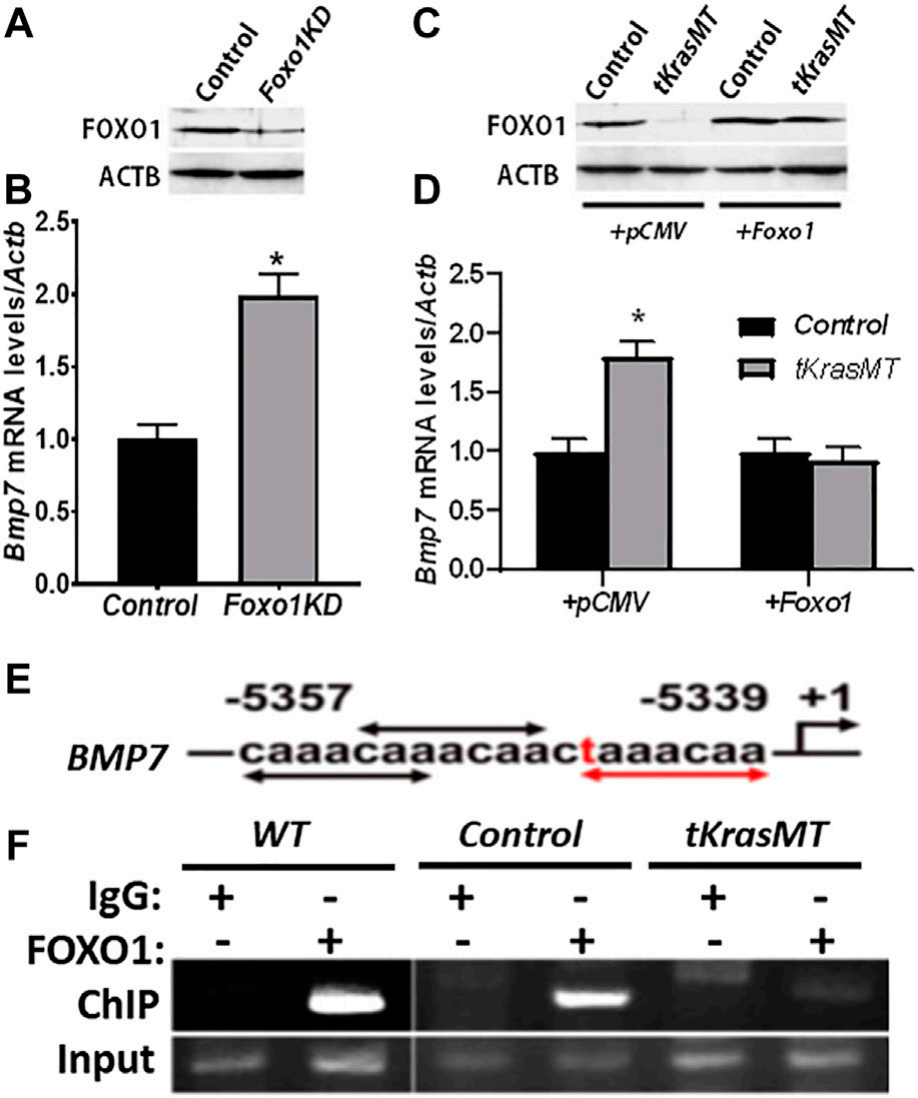
*Bmp7* is a target of KRAS/FOXO1 signaling in theca cells. **(A,B)**. Foxo1 knockdown (*Foxo1KD*) by siRNA **(A)** increases *Bmp7* expression in theca cells **(B)** (n = 3). *, *p* < 0.05 compared to the control (scrambled siRNA) group. **(C,D)**. *Bmp7* expression **(D)** in empty vector (+*pCMV*) or *pCMV-Foxo1* (+*Foxo1*) transfected *tKrasMT* theca cells **(C)**. *, *p* < 0.05 compared to the control group. **(E,F)**. Association of FOXO1 with the *Bmp7* promoter containing consensus (black arrows) and 1-mismatched (red text and arrows) FHREs **(E)** by chromatin-immunoprecipitation (ChIP) **(F)**, (n = 3).

To investigate whether *Bmp7* is indeed a target of KRAS/FOXO1 signaling in theca cells, ChIP assays were performed. The results showed that endogenous FOXO1 in theca cells was able to interact with the *Bmp7* promoter containing FHRE repeats at the -5,357 to -5,339 base-pair region, and this interaction was attenuated in *tKrasMT* theca cells (Figures 6E,F). Knockdown of *Foxo1* in theca cells by RNA interference (Figure 6A) enhanced *Bmp7* expression (Figure 6B). In contrast, *Bmp7* expression in *tKrasMT* theca cells (Figure 6C) was curtailed by *Foxo1* overexpression (Figure 6D). Therefore, *Bmp7* is a target of KRAS/FOXO1 signaling in theca cells.

## Discussion

*Kras* is expressed in ovarian theca cells in addition to oocytes and granulosa cells, which is consistent with previous reports that KRAS protein was also detected in various types of cells (Fan and Richards, 2010; Fan et al., 2012; Richards et al., 2012). KRAS is known to be essential for normal embryonic development (Koera et al., 1997) and the potential functional redundancies among three RAS proteins (Johnson et al., 1997), The loss of KRAS function in theca cells might not allow us to uncover the roles of theca KRAS in gonadal development and fertility. Therefore, a gain-of-function knock-in strategy to selectively express a constitutively active *Kras*, *Kras* ^*G12D*^ (Johnson et al., 2001), in theca cells was adopted in this study*. tKrasMT* female mice exhibited anovulation, absence of corpus luteum formation and infertility. These abnormities are different from those observed in previously reported granulosa-specific *Kras* mutants. Selective expression of *Kras* ^*G12D*^ in granulosa cells resulted in abnormal follicle growth arrest leading to premature ovarian failure and female subfertility (Fan et al., 2008b). These findings indicate that despite the universal expression of *Kras* in the ovary, KRAS in theca cells has distinct functions.

Dysregulated activation of KRAS is commonly associated with oncogenic cell transformation. Tissue-specific expression of *Kras* ^*G12D*^ in mice resulted in mammary gland, lung, pancreatic and endometrioid ovarian carcinogenesis (Fan et al., 2008b; Ludwig et al., 2016). However, ovarian tumors and hyperplasia in ovarian cells, including theca cells, were not observed in *tKrasMT* mice. Interestingly, specific activation of *Kras* ^*G12D*^ in granulosa cells or selective deletion of the tumor suppressor *Pten* in theca or granulosa cells did not induce sex cord-stromal tumorigenesis in mice (Fan et al., 2008a; Fan et al., 2008b; Lan et al., 2017). Theca and granulosa cells originate from the sex cord (coelomic epithelium) of the embryonic gonads. Ovarian cancers arising from these cell types are relatively rare and represent only approximately 5% of all human ovarian cancers (Richards et al., 2012). The results of the current study further support the idea that ovarian cells derived from the sex cord may be equipped with mechanisms that render them insensitive to tumorous transformation.

LH activation of the LHCGR in theca cells controls androgen biosynthesis (Richards and Ascoli, 2018). Hemorrhagic ovaries, anovulation and infertility observed in *tKrasMT* females are similar to that of female mice (*KiLHR* ^*D582G*^) expressing the constitutively active mutant *LHCGR* (Narayan, 2015)*.* However, unlike *tKrasMT* mice, *KiLHR* ^*D582G*^ females exhibited elevated androgen levels, enlarged ovaries and follicle development arrested at the preantral stage. Constitutive activation of KRAS signaling in theca cells did not significantly affect the ability of theca cells to produce androgens as revealed by normal responses to hCG-induced steroidogenic enzymes key for androgen biosynthesis, and adult *tKrasMT* mice maintained a comparable serum level of testosterone to that of wild-type females. Moreover, follicles at all developmental stages were present. These results suggest that the anovulatory defect in *tKrasMT* mice is not due to a dysfunctional LH/LHCGR signaling in theca cells.

In addition to androgens, a number of growth regulatory factors produced by theca cells are also important for normal follicle development and participate in the regulation of ovulation processes (Magoffin, 2005; Young and McNeilly, 2010; Richards et al., 2018). Interestingly, our data showed that *Bmp7* expression was impressively upregulated in *tKrasMT* theca cells. BMP7, a member of the transforming growth factor family, has been shown to inhibit ERK1/2 activity in an autocrine/paracrine manner (Otani et al., 2007; Wang et al., 2017; Ma et al., 2019). *Bmp7* is restrictedly expressed in theca cells of rodent ovaries, and its putative type I receptors and downstream effectors are present in granulosa/cumulus cells (Erickson and Shimasaki, 2003; Shimasaki et al., 2004; Edson et al., 2010). Studies have illustrated the inhibitory effects of BMP7 on granulosa cell differentiation (Shimasaki et al., 1999; Erickson and Shimasaki, 2003; Lee et al., 2004; Juengel et al., 2006) and hCG-induced ovulation in rats (Shimasaki et al., 1999; Lee et al., 2001) and oocyte maturation in mud crabs (Shu et al., 2016; Yang et al., 2018). In contrast, increased ovulation was observed in ewes carrying a mutation of a putative BMP7 receptor BMPR1B (Mulsant et al., 2001). Luteinization inhibition of BMP7 was demonstrated by BMP7 suppressing progesterone production while enhancing estrogen synthesis in preovulatory follicles (Shimasaki et al., 1999; Miyoshi et al., 2007; Zhang et al., 2015; Rajesh et al., 2018). Thus, our present and previous results support the notion that elevated *Bmp7* expression in *tKrasMT* theca cells is at least a cause of anovulation and the lack of corpus luteal formation displayed in *tKrasMT* females.

The LH surge plays a central role in triggering ovulation by exquisitely orchestrating a complex intraovarian network of autocrine and paracrine pathways. Fan *et al* explicitly demonstrated using knockout mouse models that ERK1/2 activity in granulosa cells was essential for ovulation (Fan et al., 2009b). It is known that the ovulatory LH surge alters the expression of various genes in granulosa cells that are necessary for ovulation (Richards, 2001; Wissing et al., 2014). Constant activation of KRAS signaling in theca cells seems to play a minimal role in the maintenance of high levels of *Npr2* expression and downregulation of *Nrip1* expression in granulosa cells to inhibit oocyte maturation and/or ovulation. It also appears to be unlikely due to the lack of *de novo* production of EGF-like factors to initiate ovulation since a hyper-response of hCG-induced *Areg* and *Btc* expression was observed. Surprisingly, constant activation of KRAS in theca cells profoundly altered the expression of *Cyp11a1*, which is a rate-limiting factor involved in the first step of progesterone synthesis, and *Cyp19a1*, which is a cardinal enzyme for estrogen production, in conterminous granulosa cells in response to hCG treatment and sustained a high serum level of estrogen. The current results provide evidence that aberrantly activated KRAS stimulates *Bmp7* expression in theca cells. The elevated theca-derived BMP7 acts in a paracrine manner on adjacent granulosa cells, resulting in ERK1/2 inhibition, *Cyp11a1* downregulation and *Cyp19a1* upregulation in response to hCG treatment. It is known that the expression of *Cyp11a1* rapidly increases after the ovulatory LH surge, whereas the expression of *Cyp19a1* rapidly decreases after the LH surge (Su et al., 2006; Wissing et al., 2014). The rapid changes in the expressions of these genes after the LH surge facilitate progesterone production by shifting estrogen synthesis to progesterone synthesis. This functional change in steroidogenesis plays a critical role in follicle rupture and the subsequent corpus luteal formation (Tanaka et al., 1991; Lydon et al., 1995; Okada et al., 2016). The completely reversal of hCG-induced expression of the *Cyp11a1* and *Cyp19a1* genes in *tKrasMT* granulosa cells may impair estrogen and progesterone signaling and lead to defective ovulation and the lack of corpus luteal formation. However, the mechanism by which KRAS/BMP7 signaling differentially regulates *Cyp11a1*, *Cyp19a1*, *Areg* and *Btc* expression in granulosa cells is required further investigation.

FOXO1 is a downstream target of many signaling pathways, including KRAS/ERK1/2 signaling, in nongonadal cells and granulosa cells (Huang and Tindall, 2007, 2011; Richards, 2018; Link, 2019). Although activation of KRAS signaling is able to regulate diverse downstream effectors, our data demonstrated that constitutive activation of KRAS signaling in theca cells specifically reduced phosphorylated and total FOXO1 protein levels. FOXO1 is a member of a subfamily of transcription factors, including FOXO1, FOXO3, FOXO4, and FOXO6. The functions of FOXO1 are controlled by its phosphorylation status. Phosphorylation of AKT and CREB in *tKrasMT* theca cells was not affected, while phosphorylation of ERK1/2 was diminished. This is possibly due to elevated expression of *Dusp6*, an ERK1/2-specific phosphatase, caused by persistent activation of KRAS, similar to the scenarios observed in granulosa cells and other cells (Camps et al., 2000; Fan et al., 2008b; Ekerot et al., 2008; Reizel et al., 2010; Donaubauer et al., 2016). Thus, reduced FOXO1 protein in *tKrasMT* theca cells is likely caused by altered ERK1/2 activity, which may trigger FOXO1 degradation (Huang and Tindall, 2011). Although *Foxo1* has been reported to be mainly expressed in granulosa cells (Ting and Zelinski, 2017), the reduced FOXO1 observed in *tKrasMT* theca cells is unlikely to be due to the potential contamination of granulosa cells. Our reasoning is that 1) *Kras* ^*G12D*^ was deleted only in theca cells, not granulosa cells, 2) theca cells were isolated using a Percoll gradient, and granulosa cell marker genes *Fshr* and *Cyp19a1* were undetectable by RT-PCR (data not shown), and 3) *Foxo1* transcripts and protein were detected in theca cells by both RT-PCR and Western blot, respectively.

FOXO1 is a transcription factor that binds FHREs to regulate gene expression, and this activity can be influenced by AKT and ERK1/2 through a mechanism of phosphorylation and nuclear exclusion of FOXO1 (Huang and Tindall, 2011; Shen et al., 2014; Link, 2019). *Bmp7* is likely a downstream target gene of FOXO1 in theca cells, since several putative FHREs were observed on the *Bmp7* promoter. More importantly, FOXO1 bound to two of the putative FHREs. FOXO1 binding was attenuated in *tKrasMT* theca cells. Furthermore, *Foxo1* impoverishment increased, whereas *Foxo1* overexpression decreased, *Bmp7* expression in theca cells. Together, our results suggest that upregulation of *Bmp7* expression in *tKrasMT* theca cells is likely through a decrease in FOXO1 to alleviate the inhibition of *Bmp7* promoter activity.

Taken together, our work provides the first *in vivo* evidence that maintaining normal KRAS activity in theca cells is essential for successful ovulation, corpus luteum formation, and female fertility. One underlying mechanism of KRAS action may be mediated through FOXO1/BMP7 signaling in theca cells. Further understanding of the regulation of FOXO1 stability, molecular and functional characterization of BMP7 controlled by KRAS signaling in ovarian theca cells, and identification of the downstream cascade(s) in granulosa cells in each ovulation event may help to determine the causes of certain types of anovulation and female infertility and to develop potential therapeutic approaches for these disorders.

## Data Availability

The original contributions presented in the study are included in the article/Supplementary Material, further inquiries can be directed to the corresponding authors.
