# Supplementary figures and images for: Aberrant activation of KRAS in mouse theca-interstitial cells results in female infertility
Source: Front Physiol. 2022 Aug 19;13:991719. doi: 10.3389/fphys.2022.991719 (PMC9437434; doi:10.3389/fphys.2022.991719)

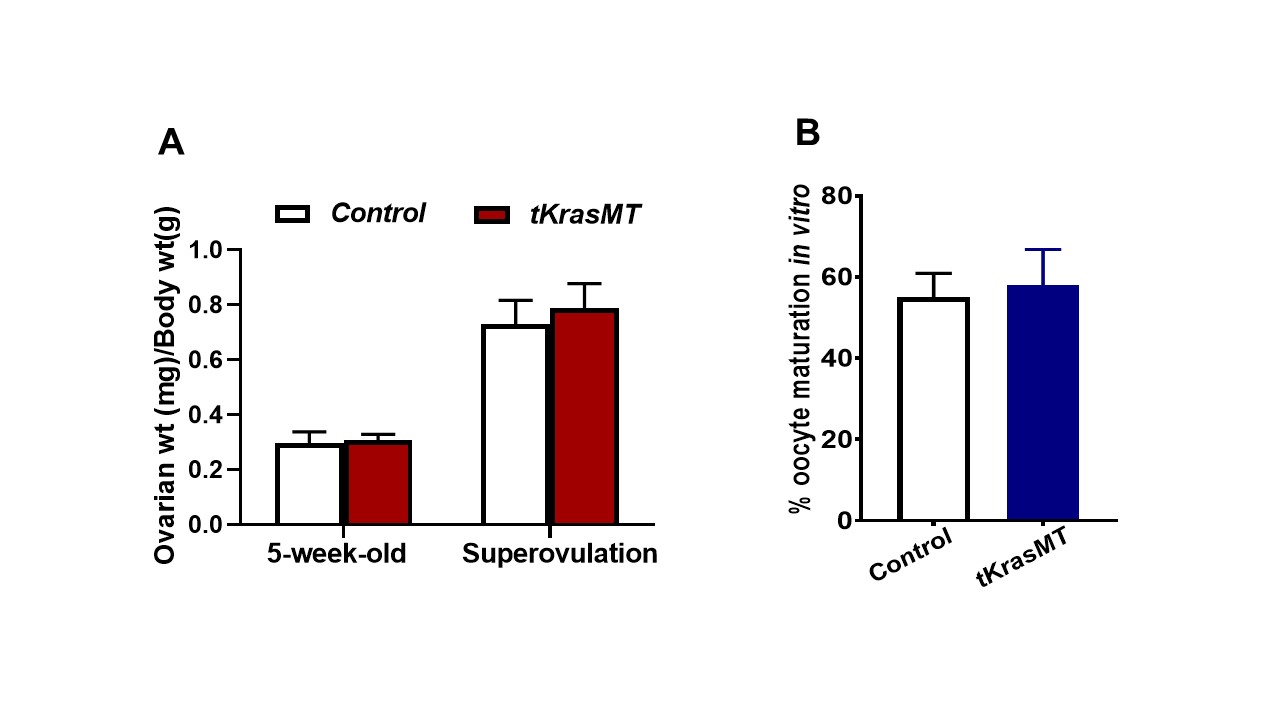

Supplement: Supplementary file 1 [file Image3.JPEG]

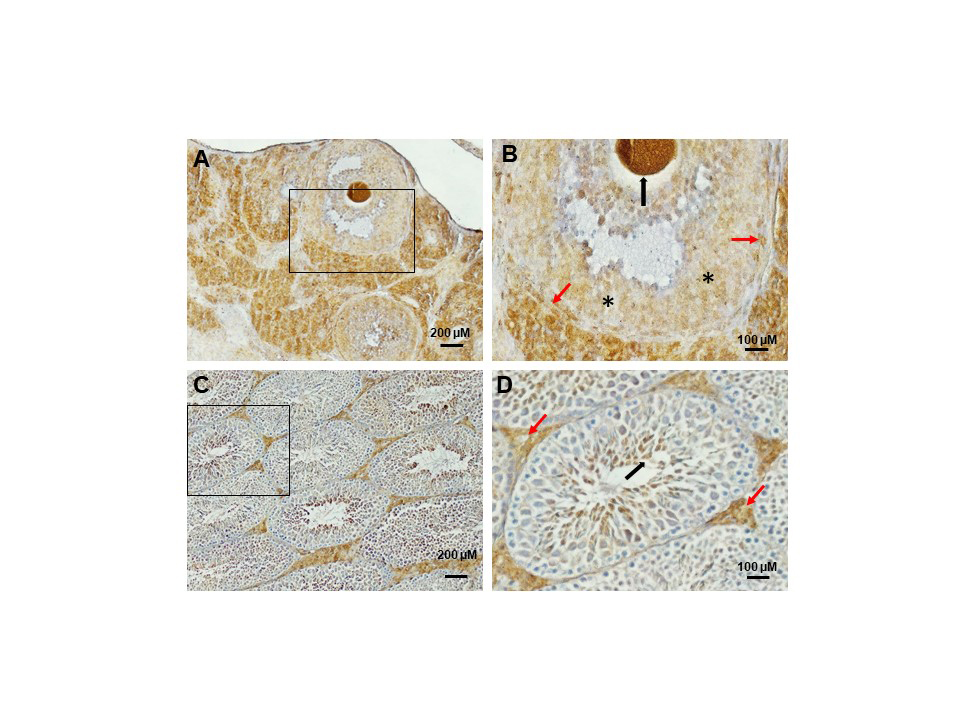

Supplement: Supplementary file 2 [file Image1.JPEG]

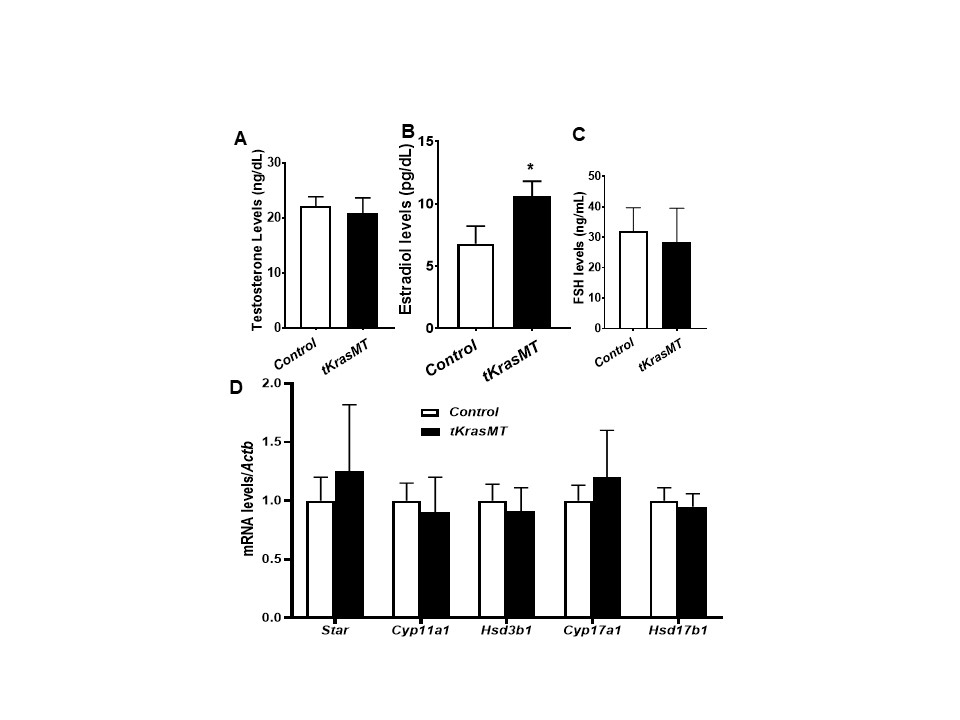

Supplement: Supplementary file 3 [file Image4.JPEG]

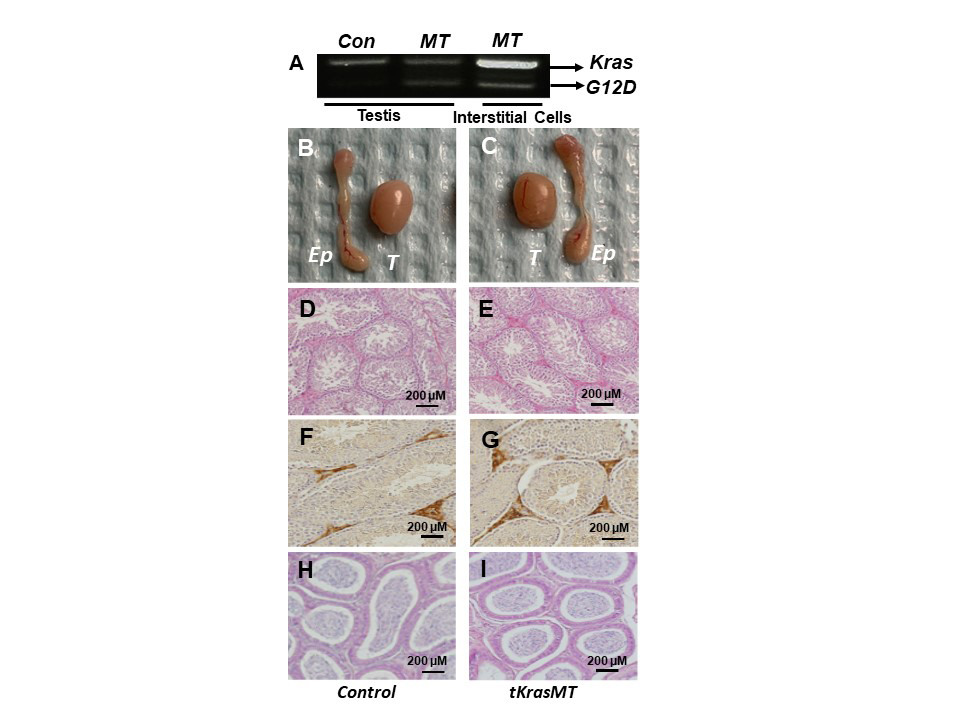

Supplement: Supplementary file 4 [file Image2.JPEG]
