# Supplementary material for: Aberrant activation of KRAS in mouse theca-interstitial cells results in female infertility
Source: Front Physiol. 2022 Aug 19;13:991719. doi: 10.3389/fphys.2022.991719 (PMC9437434; doi:10.3389/fphys.2022.991719)
Supplement: Supplementary file 5 [file DataSheet1.pdf]

Supplementary Figure legends

Fig. S1. Immunohistochemical staining of KRAS in mouse ovaries (A & B) and testes (C & D) showing that KRAS is expressed in multiple cell types in gonads, which includes theca (red arrows) and granulosa cells (*) as well as oocytes (black arrow) in the ovary (A & B), and interstitial (red arrows) and spermatogenic cells (black arrow) in the testis (C & D). The testicular sections are counterstained with hematoxylin. Boxed areas in B & D are enlarged in C & E, respectively. Representative images are from one of three mice examined.

Fig. S2. Testicular phenotype of *tKrasMT* male mice. RT-PCR demonstrates *Kras*^{G12D} expression in interstitial cells of the testis in *tKrasMT* males but not in *LslKras*^{G12D} control mice (A). Gross morphology of the testis and epididymis of *LslKras*^{G12D} controls (B) and *tKrasMT* (C) mice. Histology of H&E-stained testicular (D - G) and epididymal (H & I) sections. Immunohistochemical staining of HSD3B1 in interstitial cells of *LslKras*^{G12D} controls (F) and *tKrasMT* (G) testes.

Fig. S3. Ovarian weight (wt)/body weight (wt) of 5-week-old (n=6) and superovulated 3- to 4-week-old (n=5) *LslKras*^{G12D} (control) and *tKrasMT* females (A). Metaphase II oocytes (%) of *LslKras*^{G12D} (control) and *tKrasMT* at 24 hr culture *in vitro* (B).

Fig. S4. Serum testosterone (A), estradiol (B) and FSH (C) levels of *LslKras*^{G12D} controls and *tKrasMT* mice measured by radioimmunoassays. mRNA levels of steroid biosynthesis genes in theca cells of *tKrasMT* females determined by qRT-PCR (D). *, $P < 0.05$ compared to the control (*Lslkras*^{G12D}) group (n=6).
